# Antitumour Potential of *Gigartina pistillata* Carrageenans against Colorectal Cancer Stem Cell-Enriched Tumourspheres

**DOI:** 10.3390/md18010050

**Published:** 2020-01-12

**Authors:** João Cotas, Vanda Marques, Marta B. Afonso, Cecília M. P. Rodrigues, Leonel Pereira

**Affiliations:** 1 MARE—Marine and Environmental Sciences Centre, Faculty of Science and Technology, University of Coimbra, 3001-456 Coimbra, Portugal; jcotas@gmail.com; 2 Research Institute for Medicines (iMed.ULisboa), Faculty of Pharmacy, University of Lisboa, 1649-003 Lisboa, Portugal; vismsmarques@ff.ulisboa.pt (V.M.); mbafonso@ff.ulisboa.pt (M.B.A.); cmprodrigues@ff.ulisboa.pt (C.M.P.R.); 3 Department of Life Sciences, University of Coimbra, 3000-456 Coimbra, Portugal

**Keywords:** antitumour activity, carrageenan, colorectal cancer, cancer stem cells

## Abstract

*Gigartina pistillata* is a red seaweed common in Figueira da Foz, Portugal. Here, the antitumour potential of *G. pistillata* carrageenan, with a known variable of the life cycle, the female gametophyte (FG) and tetrasporophyte (T) was evaluated against colorectal cancer stem cell (CSC) -enriched tumourspheres. FTIR-ATR analysis of *G. pistillata* carrageenan extracts indicated differences between life cycle phases, being FG a κ/ι hybrid carrageenan and T a ʎ/ξ hybrid. Both carrageenan extracts presented IC_50_ values inferior to 1 μg/mL in HT29-derived CSC-enriched tumourspheres, as well as reduced tumoursphere area. The two extracts were also effective at reducing cellular viability in SW620- and SW480-derived tumourspheres. These results indicate that carrageenans extracted from two *G. pistillata* life cycle phases have antitumour potential against colorectal cancer stem-like cells, specially the T carrageenan.

## 1. Introduction

Seaweeds are turning into one of the most attractive natural resources of compounds that can replace chemically synthetized compounds, such as plastic and other petroleum-based products, or those from animal origin, such as collagen (gelatin). Seaweeds main structural component is a polysaccharide-based type; its main function is to maintain the structure of the cell wall, with alginic acid being the main polysaccharide produced in brown algae (Ochrophyta, Phaeophyceae), and agar (agarophytes) and carrageenan (carrageenophytes) in red algae (Rhodophyta).

The use of seaweed biopolymers is increasing in the food industry as natural gelling and emulsifier agents [1]. Carrageenans are one of the main natural texturizing agents in food applications (dairy products, jellies, pet foods, sauces) being considered safe food additives [2]. They are also used in pharmacological formulations, in cosmetics, as biomedical polymer compounds or as lubricant [2,3,4]. Overall, carrageenans principal functions in industry are as a gelling, stabilizing, and viscosity-building agent.

Diverse types of carrageenan are acquired from different species of the Gigartinales (Rhodophyta). Kappa (κ)-carrageenan is mostly acquired by extraction from the tropical seaweed *Kappaphycus alvarezii* (identified as “cottonii”, in the seaweed commercial area related to the food industry), while iota (ι)-carrageenan is mainly extracted from *Eucheuma denticulatum* (commercial denomination “spinosum”). In turn, lambda (ʎ)-carrageenan is acquired from various species from the genera *Gigartina* and *Chondrus* (commercial denomination “Irish moss”).

**Figure 1:**
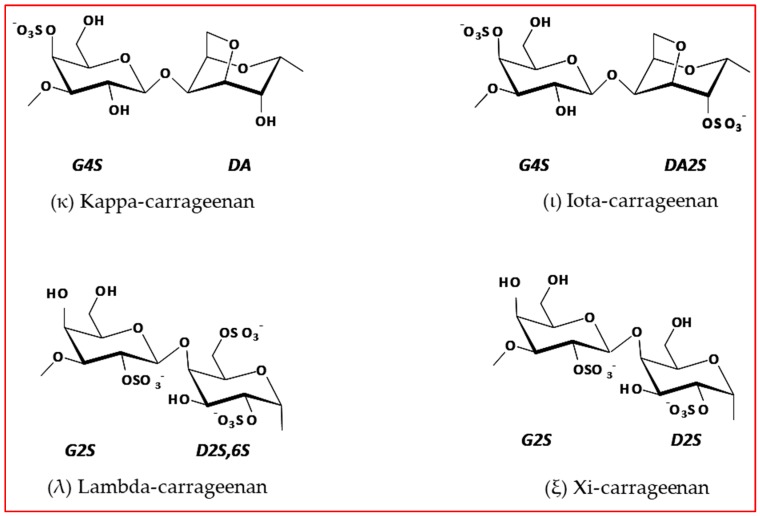
Idealized structure of the chemical units of different types of carrageenan [8].

This unique hydrocolloid consists of alternating galactose and 3,6-anhydrogalactose sugars linked by alternating α-1,3 and β-1,4 glycosidic linkages [5,6]. Carrageenans can be classified as ʎ, κ, and ι according to the number of sulphated groups by galactose unit (Figure 1), where number, chemical location, and arrangement of these groups defines carrageenan function and bioactivity power [2,7]. 

The Xi (ξ)-carrageenan, a non-commercial carrageenan, has two sulphated ester groups, minus one that is the λ-carrageenan but identical to ι-carrageenan. ξ-carrageenan is a viscous type carrageenan due to the number of sulphated esters present in the structure [8]. The k-carrageenan only has a sulphated ester group by the galactose unit.

Commonly, seaweeds do not produce these flawless and clean carrageenans, but instead a full variety of hybrid configurations of carrageenan within the species life cycle [8]. Intrinsic carrageenans always show a complex hybrid chemical conformation and are frequently a combination of galactans composed of different carrabiose types, where proportions and structures change within the species, ecological, physiological, and developmental conditions [9,10]. Hence, carrageenans are a group of polysaccharides with molecular weight varying between 30 and 5000 kDa but the average molecular weight of extracted carrageenans is between 200 and 800 kDa. [5,11]. These huge polysaccharides are nonnutritive and after ingestion have an analogous effect to dietary fibers, being extremely resistant in the digestive tract [5].

Both the Food and Drugs Administration (FDA) and the European Food Safety Agency (EFSA) have approved the commercial forms of ʎ-, κ-, and ι-carrageenans for the food industry [5,11]. In the European Union, the carrageenan application is regulated by the Commission Regulation (EU) No 231/2012, which defines that the commercial carrageenan (E 407) comprises essentially potassium, sodium, magnesium, and calcium sulphate esters of galactose and 3,6-anhydrogalactose polysaccharides [11]. Sub-chronic toxicity studies performed in rats showed that a dose of E 407 between 3400–3900 mg/kg body weight per day had no-observed-adverse-effect, fulfilling the EU specification for food level carrageenan food level [11]. On the other hand, the poligeenan, a degraded ι-carrageenan with average molecular weight of 10–20 kDa, is not authorized in food applications within the European Union area. In fact, degraded carrageenans, also known as artificial products derived from carrageenan, are associated with adverse effects [5,12]. 

Carrageenans are water soluble polymers, their solubility being determined by the type of carrageenan derivation, temperature, pH, and the counter ion in the dissolving solution, where κ-carrageenan is the most soluble. The sodium salt of κ-carrageenan is soluble in cold water, but the potassium salt is soluble only by heating. ι-carrageenan has an intermediate solubility [2]. All types of carrageenan are insoluble in organic solvents including alcohols and ketones [13].

The carrageenan extraction method plays a major role in purity control. As shown by EFSA, when carrageenans are produced by alcohol procedure, they contain approximately 90% anhydrous carrageenan, 8% moisture, and 2% inorganic salts (mainly chlorides), while those manufactured by gel press procedure contain about 77% anhydrous carrageenan, 8% moisture, and up to 15% inorganic salts [11].

**Figure 2:**
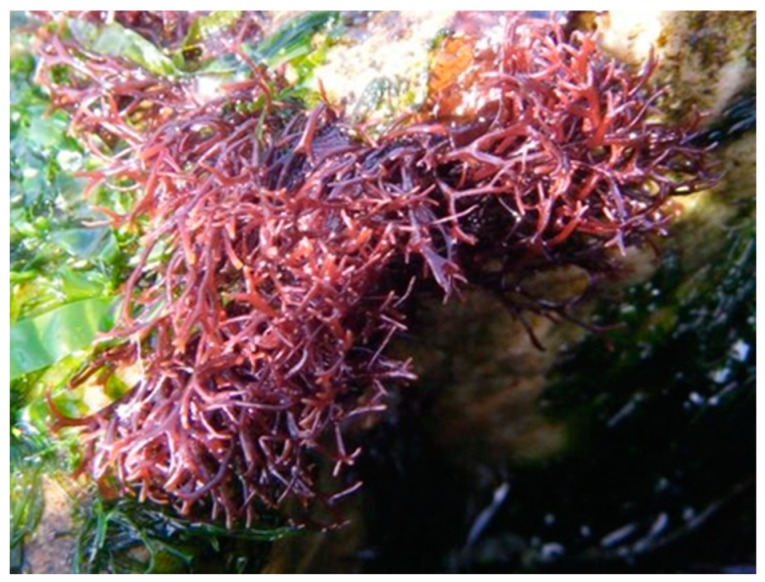
*G. pistillata* in nature, fixed in a rocky platform in Praia do Cabo Mondego, Figueira da Foz, Portugal.

*Gigartina pistillata* (Figure 2) is an edible red seaweed found in both Northeast and Southeast Atlantic and Southeast Asia, and a carrageenan resource for extraction industry. Its morphology is described as the type species of the genus *Gigartina* and their thalli are erect, up to 20 cm tall, dark-red or red-brown, cartilaginous, elastic, dichotomously branched, attached to the substratum through a small disk [14]. *G. pistillata* can show a rare presence of the heterosporic thalli (i.e., producing tetraspores and carpospores in the thalli of one specimen), despite although having an isomorphic triphasic life cycle [6]. In Gigartinaceae, the life cycle phase strongly disturbs carrageenans configuration. Gametophytic life cycle yields a κ/ι-type carrageenans, while tetrasporophytic life cycle yields a λ-type carrageenans, as shown by Pereira et al. [6,9,15,16].

The maximum carrageenan content extracted in *G. pistillata* in the work of Pereira [6,17] was obtained from female gametophyte (FG) samples, with 59.7% of dry weight in late spring; a sample of heterosporic thalli had the minimum value in late autumn, with 22.7% of dry weight.

In the last decades, the biological potential of carrageenans has been explored. Since the 1960s, carrageenan anticoagulant and antithrombotic activity has been studied with λ-carrageenan showing higher anticoagulant potential than κ-carrageenan [18]. Carrageenans have also been shown to selectively inhibit many enveloped viruses [19] and to have antioxidant properties [20]. Carrageenan antitumour potential has only been studied more recently, with several in vivo and in vitro models proposing an antiproliferative action against tumour cells [21], being compounds from marine bio-sources increasingly relevant in the quest to find new biomolecules with antitumour potential.

Cancer is undoubtedly a major cause of worldwide morbidity and mortality, with 18.1 million new cases and 9.6 million deaths estimated in 2018, and with an increasing social and economic impact. Cancer in the gastrointestinal tract plays an important role in these estimates with colorectal cancer being the third most incident (10.2%) and second most mortal (9.2%) [22]. Several studies have highlighted the role of cancer stem cells (CSCs) in colorectal cancer development, progression, and resistance to therapy [23,24,25,26,27].

The increasing evidence of the existence of CSCs has challenged the classical stochastic model of cancer dynamics and has laid the foundation for the hierarchical model of tumourigenesis, where the tumour mass is viewed as a hierarchical and unidirectional system, with CSCs in a foundational position giving rise to heterogeneous cell populations. CSCs display high self-renewal capacities, plasticity potential, high resistance to tumour microenvironment stress factors and quiescence. These properties are believed to be responsible for CSC resistance to chemotherapy, cancer relapse, and metastization [27,28,29,30]. The key role of CSCs in cancer development and recurrence opens new venues for novel therapeutic strategies based on the specific and effective targeting of this cell subpopulation. For instance, drugs that selectively inhibit CSC growth, induce cell death, force differentiation, or modulate microenvironment are promising therapeutic strategies [27,30]. By combining CSC-targeting drugs and conventional anti-cancer therapies, one can target both bulk cancer cells and CSCs, being more effective and faster in eradicating the tumour. However, it is important that noncancerous somatic stem cells are spared, particularly in tissues with high cellular turnover rates such as colon [30]. Great efforts have been made in this direction, trying to find novel drugs that specifically target CSC, particularly bioactive compounds from plant sources, where compounds such as resveratrol or curcumin show promising results [29]. In this matter, compounds from marine biosources have been less explored. Nevertheless, compounds isolated from red algae have shown some potential against CSC in an in vitro model of breast cancer [31].

Here, *G. pistillata* carrageenans from two life cycle phases, FG and tetrasporophyte (T) were analyzed by Fourier transform infrared attenuated total reflectance (FTIR-ATR) and their potential against colorectal CSC-enriched tumourspheres was explored.

## 2. Results

### 2.1. Carrageenan Extraction Yields

Through the alkali extraction method, from 1 g of dried seaweed, it was possible to obtain 17% ± 0.1 of FG carrageenan and 31% ± 3.2 of T carrageenan.

### 2.2. FTIR-ATR

**Figure 3:**
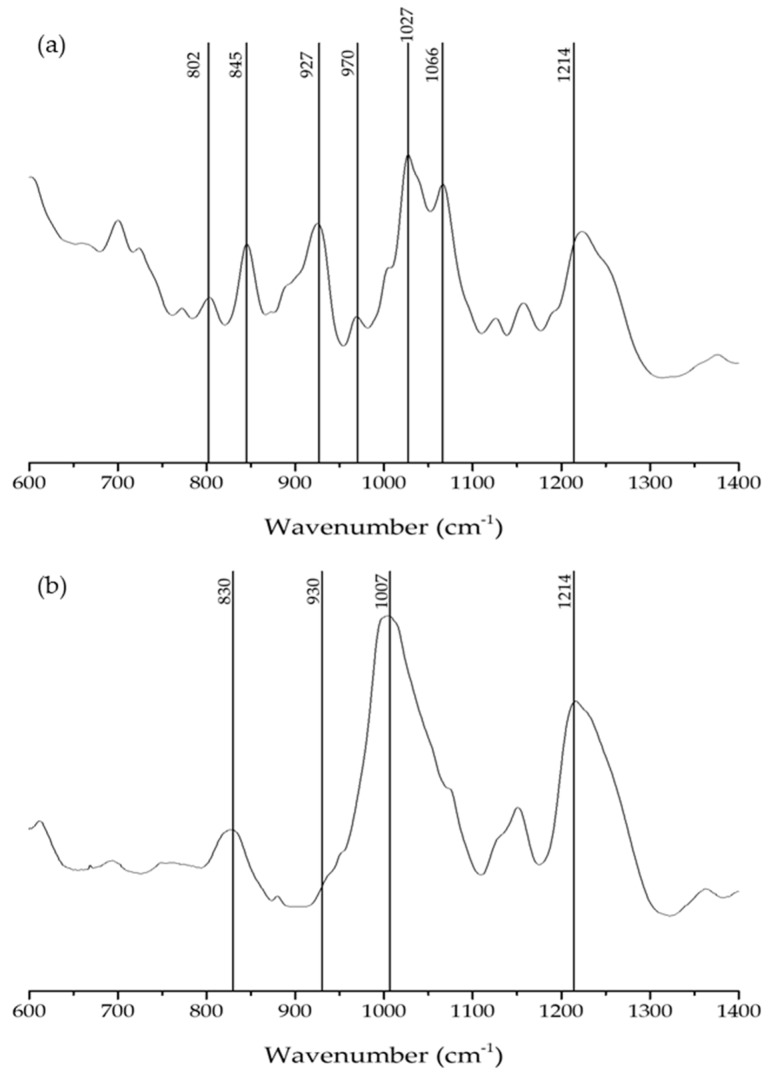
FTIR-ATR spectra of the carrageenan extracted from FG (Female gametophyte) (**a**) and T (tetrasporophyte) (**b**) *G. pistillata* life cycle.

Carrageneenan extracts from *G. pistillata* FG and T life cycles were obtained and analyzed by FTIR-ATR. This technique allowed carrageenan characterization in a fast, nondestructive manner, requiring small amounts of the sample [16]. The obtained spectra were reviewed with bibliographic support [6,8] (Figure 3 and Table 1).

In the *G. pistillata*’s FG FTIR-ATR spectrum (Figure 3a), the first band to emerge is the low intensity 802 band, corresponding to DA2S bond, indicating the presence of ι-carrageenan, while the bands at 845 and 927 cm^−1^, G4S and DA bonds, respectively, with very close intensities, indicate the presence of κ-carrageenan. A strong percentage of κ-carrageenan is also supported by the 1066 cm^−1^ band (DA). The 970 cm^−1^ band is related to the presence of galactose units.

In the *G. pistillata* T spectrum (Figure 3b) the first band appears at 830 cm^−1^, corresponding to the G/D2S, this indicates the presence of ʎ-carrageenan. The following band at 930 cm^−1^, appears as a low intensity shoulder, in opposition to the strong band observed in the FG carrageenan, the same band (the 927 cm^−1^, belonging to the same type of bond) in the spectrum presents a more noted band.

Bands at 1007 and 1214 cm^−1^ correspond to sulphated esters groups which dominate the spectrum with their very intense and broad profile, indicating a noted presence of sulphated esters groups in the T carrageenan, because T forms a nongelling carrageenan. It is noted that the 1214 cm^−1^ can be derived from Xi (ξ) carrageenan.

### 2.3. Anti-CSC Potential of Carrageenan Extracts

**Figure 4:**
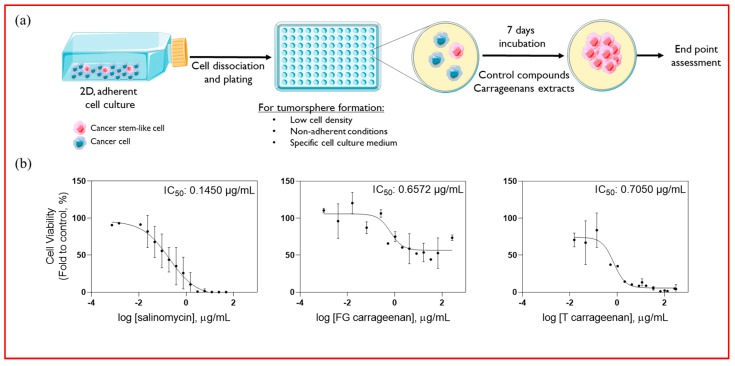
Carrageenan extracts reduce cellular viability in cancer stem cell (CSC)-enriched tumourspheres derived from HT29 colorectal cancer cell line. (**a**) Strategy for CSC-enriched tumoursphere formation; (**b**) IC_50_ determination for salinomycin, T and FG carrageenans in HT29 CSC-enriched tumourspheres. Cell viability was determined by ATP (adenosine triphosphate) metabolism assays. Results are depicted as mean ± SD, *n* = 3.

The tumoursphere forming assay [32] allows the establishment of 3D spheroids enriched in CSCs from originally 2D adherent colorectal cancer cell cultures (Figure 4a). This cellular model was applied to evaluate the potential of T and FG carrageenan extracts against CSC-enriched tumourspheres derived from colorectal cancer cell lines. In CSC-enriched tumourspheres derived from HT29 cell line, both extracts presented IC_50_ values inferior to 1 µg/mL (0.6572 and 0.7050 µg/mL for FG and T extract, respectively), which are similar to salinomycin (0.1450 µg/mL), a known CSC-targeting agent (Figure 4b) [33].

**Figure 5:**
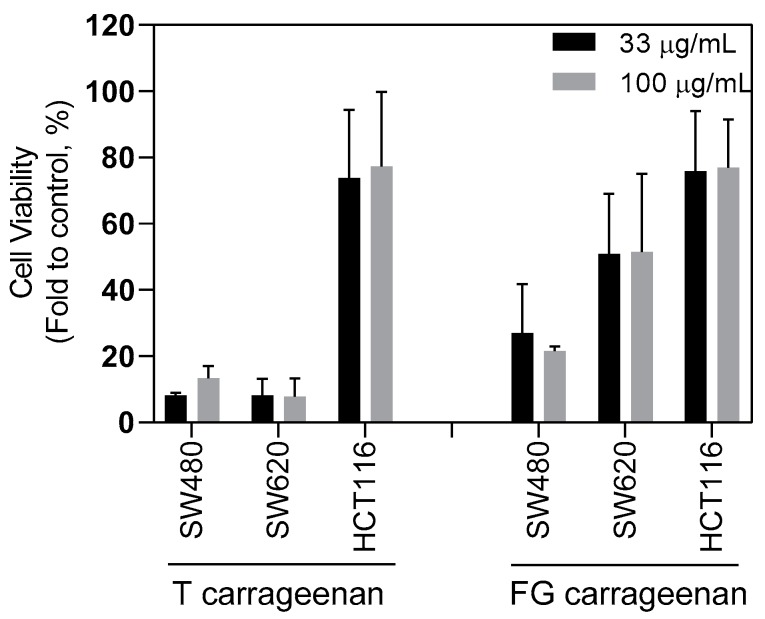
Carrageenan extracts have broad anti-CSC action. Both T and FG carrageenans impact at different extents in CSC-enriched tumourspheres derived from SW620, SW480, and HCT116 cell lines, in a dose-independent manner. Cell viability was determined by ATP metabolism assays. Results are depicted as mean ± SEM, *n* = 3.

To assess if this effect was cell line dependent, FG and T carrageenans were incubated at two different concentrations (33 and 100 μg/mL) with CSC-enriched tumourspheres derived from three different human colorectal cell lines: SW620, SW480, and HCT116 cells. T carrageenan extract markedly reduced cellular viability in SW620- and SW480-derived tumourspheres in a dose-independent manner (< 20% for both cell lines), while FG carrageenan was more effective in SW480-derived tumourspheres (< 30% for SW480 and ~50% for SW620) (Figure 5). Reduction in cellular viability was less evident in HTC116-derived tumourspheres (~75% for both T and FG carrageenans) (Figure 5).

**Figure 6:**
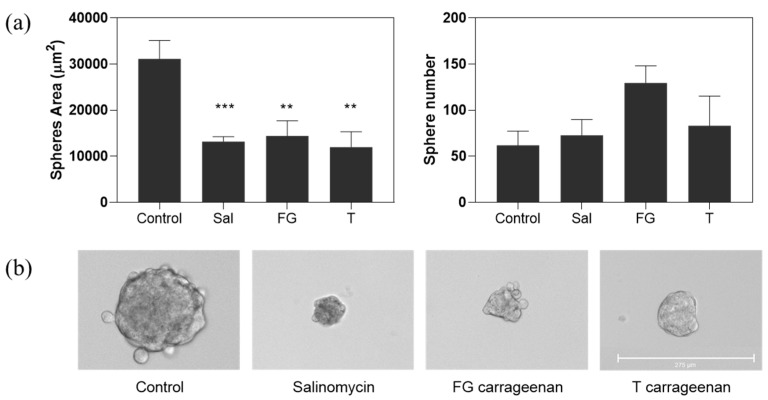
Carrageenan extracts impact on HT29-derived CSC-enriched tumoursphere formation when incubated at their respective IC_50_ dose (FG carrageenan: 0.6572 µg/mL; T carrageenan: 0.7050 µg/mL; salinomycin: 0.1450 µg/mL). (**a**) A significant reduction on tumoursphere size is observed, however tumoursphere number is not significantly altered. Results are depicted as mean ± SEM, *n* = 3. ** *p* < 0.005; *** *p* < 0.001. Sal—salinomycin. (**b**) Representative images of seven-day tumourspheres at 10× magnification. Scale bar, 275 µm.

Moreover, in HT29-derived tumourspheres, both T and FG extracts impacted on tumoursphere formation, by significantly reducing the tumoursphere area when incubated at their IC_50_ dose (*p* = 0.0017 and *p* = 0.0056 for T and FG, respectively) (Figure 6), similarly to salinomycin control (*p* = 0.0007). On the other hand, tumoursphere numbers slightly increased, particularly for FG carrageenan, although without statistical significance (Figure 6).

## 3. Discussion

Seaweed collection sites and season confer variability of the dominant generation (between gametophytes and tetrasporophytes), and reproductive cycle stage specially affects the carrageenans structure [10,17]. Here, the FG carrageenan yield (17%) was lower when compared with previous reports (59.7%) [17]. This could be explained by differences on the collection season, since in this study sampling was performed in late August and not in the Spring, which is considered the best season [17]. Concerning T carrageenan, the yield (31%) was comparable to that reported before by Amimi et al. (37%) [10].

The extracellular matrix of carrageenans resides mainly in the hydrophilic sulphated polygalactans. The existence of d-galactose and anhydrous d-galactose differentiates the highly sulphated carrageenans from the fewer sulphated agar (anhydrous-l-galactose) [34]. Analyzing the FTIR-ATR spectra of FG *G. pistillata*, strong absorption bands can be observed at ~927 cm^−1^ which is characteristic of carrageenan κ, ι, beta (β), and theta (θ), indicating the presence of C–O of 3,6-anydrogalactose (DA). Bands in the 845 cm^−1^ region (characteristic of the ι and κ carrageenan spectra, and mu (µ) and nu (ν) biological precursors of κ-carrageenan), indicate the presence of C–O–SO_3_ on C_4_ of galactose (G4S), and bands in the 970 cm^−1^ area indicate the presence of galactose (G/D), which is characteristic of κ, ι, β, and θ carrageenans spectra. However, the FG spectrum presented absorbance in the 802 cm^−1^ region indicating the presence of C–O–SO_3_ on C_2_ of 3,6-anhydrogalactose (DA2S), which is only observed in the ι and θ carrageenan spectra, meaning the presence of small amounts of ι carrageenan [8,16]. These results indicate that the FG carrageenan spectrum belongs to a κ/ι hybrid carrageenan, which is corroborated by previous results [16]. In turn, FTIR-ATR spectra of *G. pistillata* T carrageenan showed a broad band in the 830 cm^−1^ spectral region, indicating the presence of C–O–SO_3_ on C_2_ of galactose (G/D2S), which is observed in the ʎ-carrageenan spectra and λ-carrageenan precursors. In these spectra, bands in the 930 cm^−1^ region indicate the presence of 3,6-anhydrogalactose (DA), characteristic of the κ, ι, β and θ carrageenan spectra [8,16]. Therefore, this spectrum belongs to a hybrid carrageenan ʎ/ξ spectrum, mostly ʎ type. Both T and FG carrageenan spectra share strong absorption bands in the 1000–1100 and 1210–1260 cm^−1^ regions, corresponding to sulphated esters groups (S=O), which are always present in sulphated polysaccharide samples, in contrast to other vibrational bands that are characteristic of carrageenans [8]. These results are corroborated by the studies realized by Amimi [9] and Pereira [16] that proved the production of a hybrid ʎ/ξ carrageenan by *G. pistillata* T and a hybrid κ/ι carrageenan by the *G. pistillata* FG.

Sphere forming assays have been extensively used to evaluate the potential of conventional and novel molecules to target CSCs, since it has been shown that culturing 3D spheres in specific experimental conditions promotes CSC growth and propagation [29,35,36,37]. The anti-CSC potential of T and FG carrageenan extracts from *G. pistillata* was evaluated using a recently described CSC-enriched tumoursphere forming assay [32], evidencing their bioactivity particularly for T extract. Both T and FG extracts presented IC_50_ values inferior to 1 µg/mL in HT29-derived CSC-enriched tumourspheres. However, when tested in tumourspheres derived from other colorectal cancer cell lines, T extract, with ʎ/ξ hybrid carrageenans, was more effective in reducing cellular viability, particularly in SW480 and SW620 cell lines, but not in HCT116. This may be since HT29, SW620, and SW480 cell lines share genetic features such as *TP53* and *APC* mutations or chromosomal instability, which are not present in the HCT116 cell line, suggesting potential mechanisms of action. In addition, by impacting on tumoursphere formation through reducing the tumoursphere area, the potential anti-CSC action of T and FG extracts may be due to antiproliferative activity. Conversely, the increase of tumoursphere number could suggest an increase of stemness properties; however, these changes are not significant when compared to the control. Carrageenan extract from *Kappaphycus alvarezii* inhibits cell growth in several cancer cell lines, including HT29 colon cancer cell line with an IC_50_ value of 73.87 µg/mL [38]. Further, it has been demonstrated that carrageenans can delay cell cycle progression in HeLa cells, where ʎ carrageenans prolonged cell cycle by stalling in G1 and G2/M phases, therefore presenting a strong antiproliferative activity by preventing cellular division [39].

Interestingly, FITR-ATR analysis showed a presence of higher sulphate ester groups in T carrageenan compared to FG carrageenan. The quantity of sulphated esters in the carrageenan extracts may possibly be correlated with the observed antitumour effects. However, previous reports are conflicting. While Murad et al. showed that sulphated carrageenans from the red algae *Palisada perforata* (formerly *Laurencia papillosa*) induced apoptosis in a breast cancer in vitro model [40], Calvo et al. [41] reported that the presence of sulphate groups in native carrageenans diminished their cytotoxic activity in a murine mammary adenocarcinoma cell line.

Yet, several factors must be taken into consideration concerning the use of carrageenans. Although without major toxic effects [21], it has been described that λ carrageenans activate the Wnt/β-Catenin pathway in normal human colonocytes which may induce the development of intestinal polyps [42]. Moreover, one must be aware of a putative carrageenans proinflammatory potential, where reports are again conflicting. While in the past, carrageenans have been shown to induce the formation of ulcerative lesions in a guinea pig model [21] and have been used to induce paw edema in animal models [43], more recent data suggest that carrageenans do not induce the expression on proinflammatory proteins in vitro models [5]. Nevertheless, the immunomodulating effect of λ-carrageenan was explored before, suggesting that this carrageenan could be an efficient adjuvant in cancer immunotherapy by not only inhibiting cell growth but also by enhancing tumour immune response [44].

Overall, carrageenans seem to be a potential new therapeutic strategy against cancer, including therapy against CSC. Further studies are required to evaluate T and FG carrageenans toxicity, CSC-specificity, and potential mechanisms of action.

## 4. Materials and Methods

### 4.1. Reagents

Methanol was purchased from José Manuel Gomes dos Santos, Lda., Odivelas, Portugal and acetone from the Ceamed, Lda., Funchal, Portugal. Ethanol was obtained from Valente e Ribeiro. Lda., Belas, Portugal and sodium hydroxide from Sigma-Aldrich GmbH, Steinheim, Germany.

### 4.2. Seaweed Collection

The specimens of *G. pistillata* (S.G. Gmelin) Stackhouse were collected in Praia do Cabo Mondego, Buarcos, Figueira da Foz (40° 10′ 18.6″ N, 8° 53′ 44.4″ W), Portugal. Sampling was conducted in August 2018 from the sites with well-established *G. pistillata* patches and without epiphytes or degradation visible at eyesight. Once harvested, samples were stored in plastic bags for transport to the laboratory, in a cool box. All samples were washed thoroughly with filtered seawater to remove sand, and epiphytes. Specimens of *G. pistillata* were separated according to the different life cycle phases—T and FG, using a magnifying glass, washed briefly with distilled water to remove salts and dried in an air force oven (Raypa DAF-135, R. Espinar S.L., Barcelona, Spain) at 40 °C, 48 h. The dried algae were finely ground with a commercial mill (Taurus aromatic, Oliana, Spain) (≤ 1 mm) in order to render the samples uniform, and then, stored in a dark room, in a box with silica gel to reduce the humidity, at ambient temperature (± 24 °C).

### 4.3. Carrageenan Refined Alkali Extraction

Alkali extraction was preformed according to the method described by Pereira et al. [15]. The milled seaweed was weighed in a scale (Radwag WLC 1/A2, Radwag, Radom, Poland) and 1 g samples of the FG and T phases was used (*n* = 3). Before extraction, the milled seaweed material (1 g) was resuspended and pretreated with an acetone:methanol (1:1) solution in a final concentration of 1% (m/v) for 16 h, at 4 °C, to eliminate the organic-soluble fraction. The liquid solution was decanted, and the seaweed residues obtained were dried in an air force oven (Raypa DAF-135, R. Espinar S.L., Barcelona, Spain) at 40 °C before the extraction method. The samples were placed in 150 mL of NaOH (1 M) (1 g of initial seaweed: 150 mL of NaOH solution) in a hot water bath system (GFL 1003, GFL, Burgwedel, Germany), at 85–90 °C, for 3 h. The solutions were hot filtered, twice, under vacuum, through a cloth filter supported in a Buchner funnel and a Kitasato flask. The extract was evaporated (rotary evaporator model: 2600000, Witeg, Germany) under vacuum to one-third of the initial volume. The carrageenan was precipitated by adding the warm solution to twice its volume of 96% ethanol. The precipitated carrageenan was washed with ethanol, 48 h at 4 °C, before drying in an air force oven. After drying, the carrageenan was maintained in a close air sample flask stored in a dark and no humidity local, at room temperature before the antitumour and FTIR-ATR assay. For biological assays, the dried carrageenans extracts were grinded and dissolved in ultra-pure, sterile water at a final concentration of 5 mg/mL.

### 4.4. FTIR-ATR Assay

For FTIR-ATR analysis, the T and FG dried carrageenan extracts were milled using a commercial mill (Taurus aromatic, Spain) to obtain a fine powder, which was subjected to direct analysis. FTIR-ATR spectra were recorded on an IFS 55 spectrometer, using a Golden Gate single reflection diamond ATR system, with no need for sample preparation, since these assays only required dried samples, according to Pereira et al. [6]. All spectra are the average of two independent measurements with 128 scans, each at a resolution of 2 cm^−1^.

### 4.5. Cell Culture

Human colorectal carcinoma cell lines HT29, HCT116, SW620, and SW480 were obtained from ECACC (Porton Down, UK). Cell lines were cultured under adherent conditions in RPMI (HT29), McCoy’s 5A (HCT116) and Dulbecco’s modified Eagle’s medium (DMEM; SW620 and SW480), all supplemented with 10% (*v*/*v*) heat-inactivated fetal bovine serum (FBS) and 1% (*v*/*v*) antibiotic/antimycotic solution (all from Gibco, Thermo Fisher Scientific, Paisley, UK). All cell cultures were maintained at 37 °C under a humidified atmosphere of 5% CO_2_ (HeraCell 150i CO2 incubator, Thermo Fisher Scientific, Paisley, UK).

### 4.6. Tumoursphere Forming Assay

For the generation of tumourspheres, cell lines were grown as previously described [32]. Briefly, cells were plated at low density (100 and 500 cells/well in 96- and 24-well plates, respectively), in ultra-low attachment plates in a serum-free DMEM/F12 medium supplemented with 1% penicillin-streptavidin, 1% nonessential amino acids, 1% sodium pyruvate, 2% B27 supplement, 1% N2 supplement, 40 ng/mL recombinant human epidermal growth factor (all from Gibco), 4 µg/mL heparin (Biochrom GmbH, Berlin, Germany), and 20 ng/mL recombinant human basic fibroblast growth factor (Peprotech, London, UK).

### 4.7. IC_50_ Determination

To quantitatively assess the carrageenan-extract inhibitory potency in CSC-enriched tumourspheres, their half maximal inhibitory concentration (IC_50_) was determined through a 10-point dose-response curve. Briefly, HT29 cells were plated in 96-well plates as described above and treated with carrageenan extracts (concentrations ranging from 300 to 0.015 μg/mL), positive control (salinomycin (Sigma-Aldrich, St. Louis, MI, USA); concentrations ranging from 13 to 0.023 μg/mL), or vehicle control (DMSO). Following seven days of incubation, cell viability was assessed based on measurement of ATP metabolism using CellTiter-Glo™ luminescent cell viability assay (Promega, Madison, WI, USA). Luminescence signal was recorded using a GloMax^®^-MultiDetection System (Promega). IC_50_ determination was preformed using GraphPad Prism 8.0.2. software.

### 4.8. Validation of CSC-Targeting Action

To assess whether the carrageenan extracts have a generic effect on colorectal CSCs or whether this is a cell line dependent effect, they were tested on CSC-enriched tumourspheres from other colorectal cell lines. Hence, HCT116, SW480, and SW620 cell lines were cultured in 96-well plates as described above to promote CSC-enriched tumoursphere formation and treated with carrageenan extracts (100 and 33.3 μg/mL), positive control (salinomycin, at 0.751 μg/mL), or vehicle control (DMSO). Following seven days of incubation, cell viability was assessed based on measurement of ATP as described before.

### 4.9. Impact on Tumoursphere Formation

The anti-CSC effect of the carrageenan extracts was further validated by assessing their impact on tumoursphere formation. Hence, HT29 cells were plated in 24-well plates as described before and treated with hit carrageenan extracts, positive control (salinomycin) all at their IC_50_ dose, and vehicle control (DMSO). After seven days of incubation, tumoursphere images were acquired through brightfield microscopy with Invitrogen EVOS^TM^ FL Auto2 imaging system (Invitrogen, Thermo Fisher Scientific). Tumourspheres number and area were determined using ImageJ analysis software (version 1.52a, NIH, USA). Data analysis was preformed using GraphPad Prism 8.0.2. software. A *p*-value inferior to 0.05 was considered significant.

## 5. Conclusions

This work shows that the carrageenans extracted from the two *G. pistillata* life cycle phases, particularly the T carrageenan, have potential against colorectal cancer CSC-like cells. This could be explained by the higher sulphated ethers content in T carrageenan (ʎ/ξ) comparatively with the FG carrageenan (κ/ι) with support by the identification via FTIR-ATR. Little is known about the interaction between CSCs and carrageenan, and how this can affect human colorectal cell function. Hence, further studies are needed to understand the carrageenan anti-CSC mechanism of action, particularly for the T carrageenan.

## Figures and Tables

**Table 1:** FTIR-ATR bands identification and characterization of the *G. pistillata* FG and T life cycle.

Wavelength Numbers (cm^−1^)	Chemical Structure	Bonds/Assignments	Life Cycle Presence
802	C–O–SO_3_ on C_2_ of 3,6-anhydrogalactose	DA2S	FG
830	C–O–SO_3_ on C_2_ of galactose	G/D2S	T
845	C–O–SO_3_ on C_4_ of galactose	G4S	FG
927	C–O of 3,6-anydrogalactose	DA	FG
930	C–O of 3,6-anydrogalactose	DA	T
970	Galactose	G/D	FG
1007	S=O	sulphated esters	T
1027	S=O	sulphated esters	FG
1066	C–O of 3,6-anydrogalactose	DA	FG
1214	S=O	sulphated esters	FG/T
